# Functional expression of mitochondrial K_Ca_3.1 channels in non-small cell lung cancer cells

**DOI:** 10.1007/s00424-022-02748-x

**Published:** 2022-09-24

**Authors:** Etmar Bulk, Luca Matteo Todesca, Magdalena Bachmann, Ildiko Szabo, Marius Rieke, Albrecht Schwab

**Affiliations:** 1 Institute of Physiology II, University of Münster, 48149 Münster, Germany; 2 Department of Biology, University of Padova, Padua, Italy

**Keywords:** K_Ca_3.1, Mitochondria, ROS, NSCLC

## Abstract

Lung cancer is one of the leading causes of cancer-related deaths worldwide. The Ca^2+^-activated K^+^ channel K_Ca_3.1 contributes to the progression of non-small cell lung cancer (NSCLC). Recently, K_Ca_3.1 channels were found in the inner membrane of mitochondria in different cancer cells. Mitochondria are the main sources for the generation of reactive oxygen species (ROS) that affect the progression of cancer cells. Here, we combined Western blotting, immunofluorescence, and fluorescent live-cell imaging to investigate the expression and function of K_Ca_3.1 channels in the mitochondria of NSCLC cells. Western blotting revealed K_Ca_3.1 expression in mitochondrial lysates from different NSCLC cells. Using immunofluorescence, we demonstrate a co-localization of K_Ca_3.1 channels with mitochondria of NSCLC cells. Measurements of the mitochondrial membrane potential with TMRM reveal a hyperpolarization following the inhibition of K_Ca_3.1 channels with the cell-permeable blocker senicapoc. This is not the case when cells are treated with the cell-impermeable peptidic toxin maurotoxin. The hyperpolarization of the mitochondrial membrane potential is accompanied by an increased generation of ROS in NSCLC cells. Collectively, our results provide firm evidence for the functional expression of K_Ca_3.1 channels in the inner membrane of mitochondria of NSCLC cells.

## Introduction

Non-small cell lung cancer (NSCLC) is one of the leading causes of cancer-related death worldwide. The 5-year overall survival rate is low (19%) because NSCLC is often unresectable at diagnosis and (becomes) resistant to therapy [13]. Today, treatment options also depend on the genetic status of the patients and include, among others, the targeting of driver mutations of specific genes (e.g., EGFR or KRAS [18, 19, 49]). During the last decades, mitochondria became interesting targets in the treatment of cancer and other diseases [4, 11, 32] because of their important role in cellular proliferation and apoptosis [16, 35]. Mitochondria are crucial players controlling the intrinsic apoptotic pathway by regulating the release of pro-apoptotic factors such as cytochrome c [47]. Mitochondria are the main sources for the generation of reactive oxygen species (ROS), which are responsible for the (dys-) regulation of several mechanisms in almost all cells, including cancer cells. At low levels, ROS act as an important regulator of cell signaling and Ca^2+^ homeostasis. However, at high levels, ROS can result in oxidative cell damage and thus can lead to apoptosis or cell death [5]. The release of ROS is caused by a hyperpolarization of the inner mitochondrial membrane potential [33, 48, 50]. This, in turn, correlates with the release of cytochrome c, which triggers apoptosis [17].

There is firm evidence that mitochondrial ion channels contribute to cancer hallmarks so that their inhibition can be exploited therapeutically [2, 22, 23, 30, 42]. For example, blocking the mitochondrial voltage-gated K^+^ channel K_V_1.3 not only reduces the tumor size in orthotopic mouse models of melanoma and pancreatic ductal adenocarcinoma (PDAC) but also kills primary chronic B-lymphocytic leukemia tumor cells [22] and reduces clinical signs of B-CLL in a genetic mouse model of the disease [37]. Besides K_V_1.3, other ion channels have been identified in mitochondria of human cancer cells such as the Ca^2+^-activated K^+^ channel K_Ca_3.1. K_Ca_3.1 channels have been localized in mitochondria of Hela cells, colon cancer cells, melanoma cells, and PDAC cells [21, 27, 34]. Further studies provided evidence that pharmacological inhibition of K_Ca_3.1 channels by membrane-permeant drugs that can reach the mitochondria contributes to apoptosis in hepatocellular cancer HepG2 cells, malignant glioma cells [1, 25], as well as melanoma cells [3].

Previously, we revealed an increased ICAM-1 dependent adhesiveness of NSCLC cells to endothelial cells when K_Ca_3.1 channels are pharmacologically inhibited [7]. Since an up-regulation of ICAM-1 can be induced by the generation of reactive oxygen species [10, 26], we asked the question of whether K_Ca_3.1 channels are expressed in mitochondria and whether blocking them could induce the generation of ROS in NSCLC cells.

## Material and methods

### Cell culture

All cell lines were incubated at 37 ℃ and 5% CO_2_. The highly aggressive A549 lung adenocarcinoma cells (generated by Hascher et al. [14]) were cultured in DMEM medium (Dulbecco’s modified Eagle’s medium, Sigma-Aldrich) supplemented with 10% fetal bovine serum (FBS Superior, Biochrom GmbH, Germany). The human lung carcinoma cell lines H1299 (cell number CRL-5803™) and H1975 (cell number CRL-5908™), obtained from American Type Culture Collection ATCC, were grown in RPMI-1640 medium (#R8758, Sigma-Aldrich, Germany) containing 10% fetal bovine serum.

### Isolation of mitochondria

Mitochondria from NSCLC cells were isolated using the Qproteome™ Mitochondria Isolation Kit (#ID: 37,612, QIAGEN, Germany) according to the manufacturer’s instructions. For further usage, the mitochondria were resuspended in the Mitochondria Storage Buffer provided with the isolation kit.

### Western blot analysis

For the production of whole cell lysates, cells were lysed in RIPA buffer (50 mmol/l Tris, 150 mmol/l NaCl, 0.1% SDS, 0.5% sodium deoxycholate, 1% NP-40, and protease inhibitors from Roche, Germany). Isolated mitochondria (resuspended in Mitochondria Storage Buffer) were used directly. Before gel loading, the mitochondria as well as the whole cell lysates were mixed with a 5 × SDS-Page sample buffer (0.225 M Tris–Cl pH 6.8, 50% glycerol, 5% SDS, 0.25 M DTT, and 0.05% bromphenol blue) and boiled at 95 ℃ for 5 min. After gel loading and transfer to a PVDF membrane (#1,620,264, Bio-Rad, Germany), the membrane was blocked in Tris-buffered saline (TBS containing 5% skim milk and 0.05% Tween) for 1 h. We used the following antibodies: rabbit anti-K_Ca_3.1 (1:500, #AV35098, Sigma-Aldrich), rabbit anti-BAK (1:200, #AHP2279, Bio-Rad), and mouse anti-α-tubulin (1:5000, #6199, Sigma-Aldrich) as primary antibodies and goat anti-mouse and goat anti-rabbit (1:10,000, Sigma-Aldrich) as secondary antibodies.

### *Localization of K*_*Ca*_*3.1 channels in mitochondria of NSCLC cells using immunofluorescence*

Cells were seeded on glass cover slips, placed in 12-well dishes, and incubated overnight under cell culture conditions. The next day, cells were incubated with 200 nM MitoTracker™ Orange CMTMRos (Thermo Fisher Scientific, USA) at 37 ℃ in HEPES-buffered Ringer’s solution (in mmol/l: NaCl 122.5, KCl 5.4, CaCl_2_ 1.2, MgCl_2_ 0.8, HEPES 10, and D-glucose 5.5 at pH 7.4) for 45 min. For simplicity, the solution will hereafter be referred to as Ringer’s solution. Then cells were washed 3 × with ice cold PBS and fixed in 3.5% paraformaldehyde (in PBS) at 4 ℃ for 1 h. The cells were washed again 3 × with PBS and incubated in a 100 mmol/l glycine/PBS solution for 10 min. Afterwards, cells were permeabilized in PBS containing 0.1% Triton X-100 and 1% SDS for 10 min. Additional washing cells were blocked for 30 min with goat serum (10% in PBS) and incubated with a rabbit anti-K_Ca_3.1 antibody (1:500; #AV35098, Sigma-Aldrich) at room temperature for 1 h. Subsequently, cells were washed and incubated in the dark at room temperature with a secondary antibody (1: 200; goat anti-rabbit labeled with Alexa Fluor 488, Invitrogen, Carlsbad, USA) for 45 min. Finally, cells were washed 3 × in PBS, fixed in 3.5% paraformaldehyde for 10 min, and covered with fluorescence mounting medium (Dako, #S3023, USA). Two images per cell were taken using a ZEISS microscope Axiovert 200, linked to a digital camera (Model 9.0, RT-SE-Spot, Visitron Systems, Germany). The camera was controlled by MetaVue software (Visitron, Germany). The image for the detection of the mitochondria was recorded using a TRITC filter (546 nm excitation, 590 nm emission); detection for K_Ca_3.1 channels was carried out by using a FITC filter (490 nm excitation, 525 nm emission). For the following analysis, the two images were merged via the MetaFluor Software and five regions per cell (100 × 100 pixels) were chosen for localizing K_Ca_3.1 channels in mitochondria.

Using a line-scan tool, a line was placed over each channel. To determine the co-localization of K_Ca_3.1 channels in mitochondria, only those curves were chosen where the optical centers, e.g., the maxima of the curves of FITC and TRITC channels, were lying on top of each other and differed on the X-axis by not more than one pixel (= 60 nm). Another criterion was that only those dots (representing K_Ca_3.1 channels) were chosen whose apparent size was < 5 pix (equal to ~ 300 nm; [28]). For analyzing the co-localization, we counted those K_Ca_3.1 channels whose optical centers matched those of the mitochondria (TRITC). Their numbers were set in relation to the total number of K_Ca_3.1 channels in that area (100 × 100 pixels) or to the area covered by the mitochondria. We analyzed at least 30 cells for each cell line.

### Mitochondrial membrane potential measurements

The mitochondrial membrane potential was measured using the cationic fluorescent dye tetramethylrhodamine methyl ester (TMRM; #T668, Thermo Fisher). In the case of A549 cells, 7 × 10^4^ cells were seeded on glass coverslips (pre-coated for 30 min with 0.1% poly-L-lysine at room temperature) in 35 mm cell culture dishes and cultured overnight. On the following day, cells were incubated with 25 nM TMRM in HBSS at 37 ℃ in the dark. After 20 min, the TMRM concentration was reduced to 5 nM, and 4 µM cyclosporine H was added to avoid extrusion of TMRM from cells during measurements, which could mask an eventual hyperpolarization (cyclosporine H blocks the multi-drug resistance transporters (MDR) in the mitochondria [31, 36]). The glass coverslips were placed in an environmental chamber (37 ℃ and 5% CO_2_) on the stage of a Leica SP5 confocal microscope, and images were acquired in 5 min intervals. The recording started with a 10 min control period. Thereafter, senicapoc (30 µM) or Maurotoxin (20 nM) were added, and images of the same visual field were taken for another 30 min. At the end of each experiment, 1 μM of the mitochondrial oxidative phosphorylation uncoupler, FCCP (carbonyl cyanide-p-trifluoromethoxyphenylhydrazone), was added, and additional images were taken in 5 min intervals for a period of 10 min. The measurement of the mitochondrial membrane potential of H1299 cells was performed very similarly for the A549 cells. Briefly, 2.5 × 10^5^ cells were seeded on 35 mm glass-bottom dishes and cultured overnight. On the next day, H1299 cells were stained with 25 nM TMRM in Ringer’s solution for 20 min at 37 ℃ in the dark. Then, the solution was changed to 5 nM TMRM, and the dish was mounted to a ZEISS Axiovert 200 microscope equipped with a heated stage (37 ℃) and a SPOT RT SE Monochrome CCD Scientific Digital Camera System (Visitron Systems, Germany). The recording and the addition of senicapoc or Maurotoxin was performed identically as to experiments with the A549 cells.

### Determining ROS levels

We measured superoxide levels in lung cancer cells by using the mitochondrial superoxide indicator MitoSOX™ Red (#M36008, Thermo Fisher Scientific, USA). Briefly, cells were seeded on glass-bottom dishes. The next day, cells were pre-incubated in Ringer’s solution. Afterward, the cells were stained in 10 µM MitoSOX containing Ringer’s solution at 37 ℃ for 10 min. Thereafter, cells were washed 3 × with Ringer’s solution. Then, either TRAM-34 (10 µM), senicapoc (30 µM), or Antimycin A (2 µM; #A8674, Merck/Sigma-Aldrich, Germany) was added, and the dish was mounted on the heated stage (37 ℃) of a ZEISS Axiovert 200 microscope equipped with a SPOT RT SE Monochrome CCD Scientific Digital Camera System (Visitron Systems, Germany). Images were taken in 2 min intervals for a period of 30 min. Quantification of the background-corrected cellular fluorescence intensity (arbitrary units) was performed using the free imaging software ImageJ.

### Statistical analysis

In general, experimental data are shown as mean ± SEM. The mean values of more than two groups were tested using two-way ANOVA, followed by Bonferroni’s or Dunnett’s multiple comparisons test. A *p* < 0.05 (*) was considered as statistically significant.

## Results

### *K*_*Ca*_*3.1 channels are expressed in mitochondria of NSCLC cells*

**Fig. 1:**
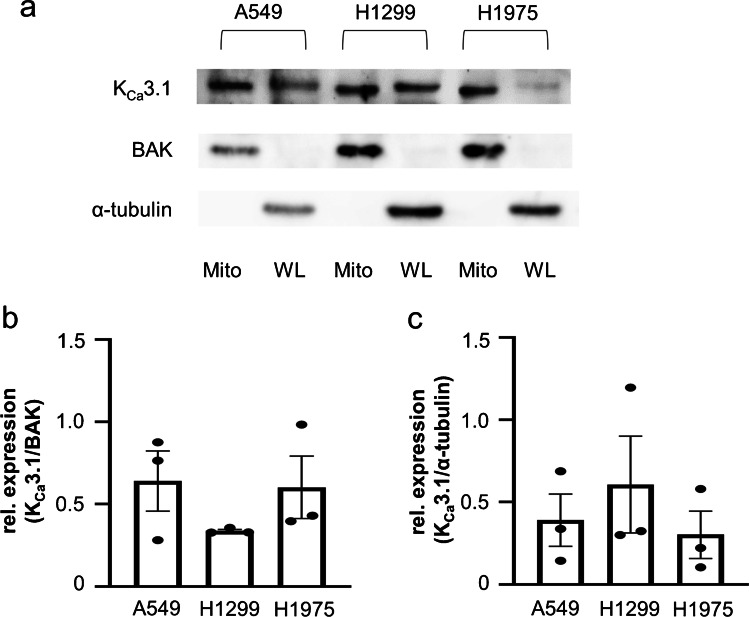
**K**_**Ca**_**3.1 channels are expressed in mitochondria isolated from NSCLC cells.** (**a**) K_Ca_3.1 protein expression in isolated mitochondria (Mito) and whole cell lysates (WL) of A549, H1299, and H1975 cells. Mitochondrial isolation was confirmed by detecting the mitochondrial marker protein BAK. An antibody against α-tubulin was used as a positive loading control for whole cell lysates (*N* = 3). (**b**) Summary of the densitometric analyses from mitochondrial lysates (normalized to BAK) and (**c**) from whole cell lysates (normalized to α-tubulin)

We applied Western blotting to provide the first evidence for the expression of K_Ca_3.1 channels in mitochondria of the NSCLC cell lines A549, H1299, and H1975 that have a different genetic backgrounds with respect to their oncogenic driver mutations. The H1975 cell line is resistant to erlotinib, a tyrosine kinase inhibitor that improves the survival of patients with epidermal growth factor receptor (EGFR)-mutated non-small cell lung cancer [46]. H1299 cells harbor a p53 mutation that expresses no wild-type p53 [44]. The A549 cell line has none of these mutations [20]. Whole-cell lysates were taken from these three different NSCLC cell lines. In parallel, mitochondria from these cell lines were isolated. Analyses revealed a band of the expected size of K_Ca_3.1 channels of ~ 50 kDa in all cell lysates and in their corresponding mitochondrial fractions (Fig. 1a and Supplementary information). We used BAK and α-tubulin as loading controls for mitochondrial and whole cell lysates, respectively. Interestingly, mitochondrial K_Ca_3.1 channels appeared to have a slightly smaller molecular weight than plasma membrane channels. Such a difference in molecular weight was also seen for several K_V_1 channels [8]. Densitometric analysis shows that K_Ca_3.1 channels are expressed in mitochondria isolated from all three NSCLC cell lines (Fig. 1b). Figure 1c shows the expression of K_Ca_3.1 channels in all whole cell lysates of the NSCLC cell lines.

### *Localization of K*_*Ca*_*3.1 channels in mitochondria of NSCLC cells*

**Fig. 2:**
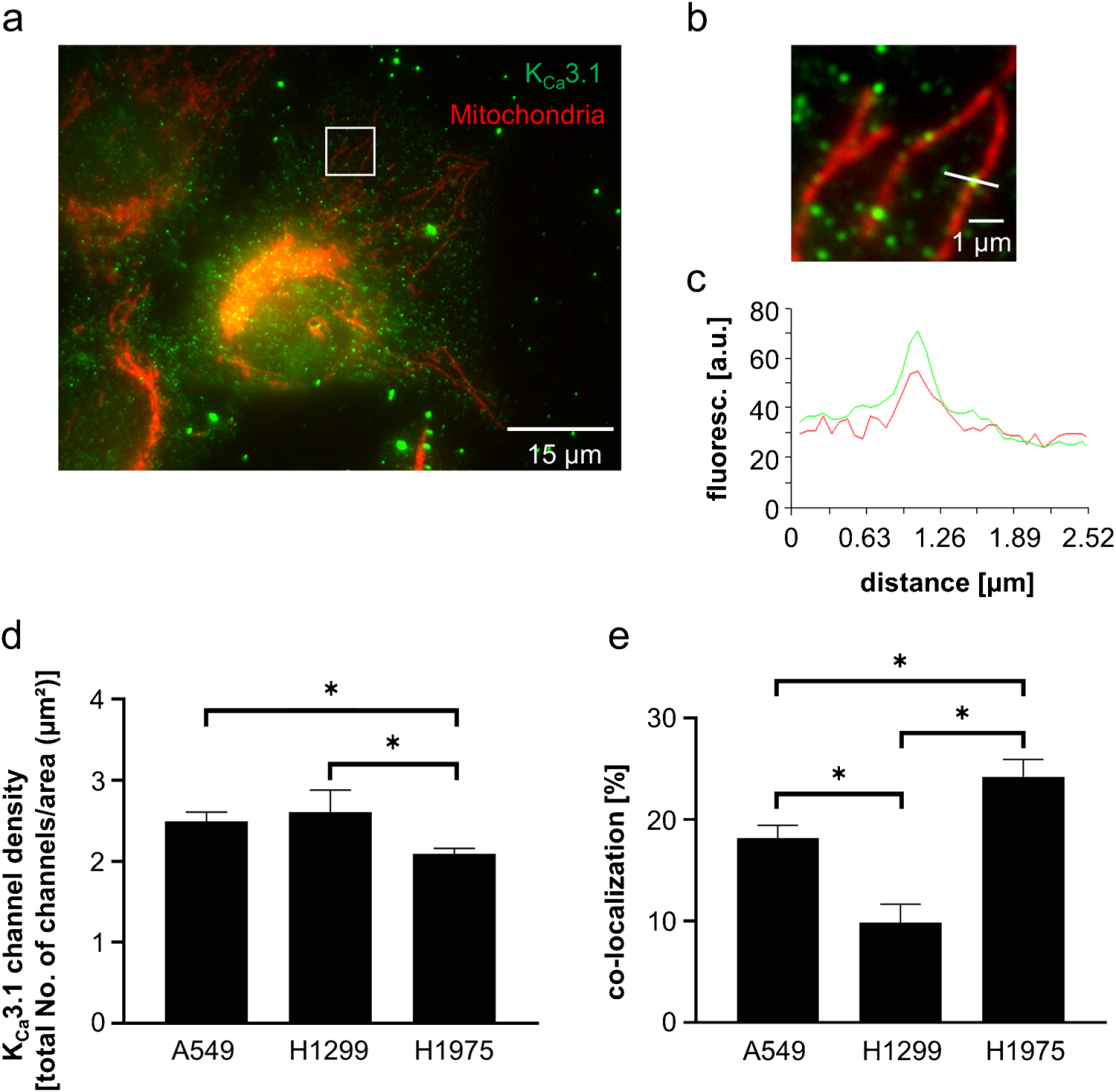
**Co-localization of K**_**Ca**_**3.1 channels and mitochondria in NSCLC cells.** (**a**) Immunofluorescence image of the lung cancer cell line H1975, co-stained for mitochondria (red) and K_Ca_3.1 channels (green). The square represents the enlarged section shown in (**b**). (**c**) Intensity profile along the white line shown in (**b**). A K_Ca_3.1 channel (green dot) colocalizes with a mitochondrium (red). (**d**) K_Ca_3.1 channel density of 5 regions per cell. Mean values of total numbers from at least 12 different cells are displayed. The statistical analysis was performed using two-way ANOVA, followed by Bonferroni’s multiple comparisons test. (**e**) Percentage of the total number of K_Ca_3.1 channels localized in mitochondria in different NSCLC cell lines. We analyzed at least 12 different cells per cell line. For the statistical analysis, a two-way ANOVA was performed, followed by Bonferroni’s multiple comparisons tests

Based on co-staining of mitochondria by using the MitoTracker™ Orange CMTMRos combined with immunofluorescence using a rabbit anti-K_Ca_3.1 antibody, we localized K_Ca_3.1 channels in mitochondria (Fig. 2a-c). The highest number of K_Ca_3.1 channels could be observed in H1299 cells (2.6 ± 0.27 total number of channels/µm^2^) and A549 cells (2.5 ± 0.12; Fig. 2d). The density of K_Ca_3.1 channels in H1975 cells is slightly lower (2.1 ± 0.07). However, when normalized to the total number of K_Ca_3.1 channels in the fields of observation H1975 cells have the highest degree of K_Ca_3.1 channel localization in mitochondria (24.0 ± 1.7%) (Fig. 2e). This percentage is lower in A549 cells (18.0 ± 1.3%) and H1299 cells (10.0 ± 1.8%).

Taken together, the results obtained by immunofluorescence are consistent with those on the protein level by Western blotting. Both methods reliably detected mitochondrial K_Ca_3.1 channels in three different NSCLC cell lines.

### *K*_*Ca*_*3.1 channel regulates the mitochondrial membrane potential of NSCLC cells*

**Fig. 3:**
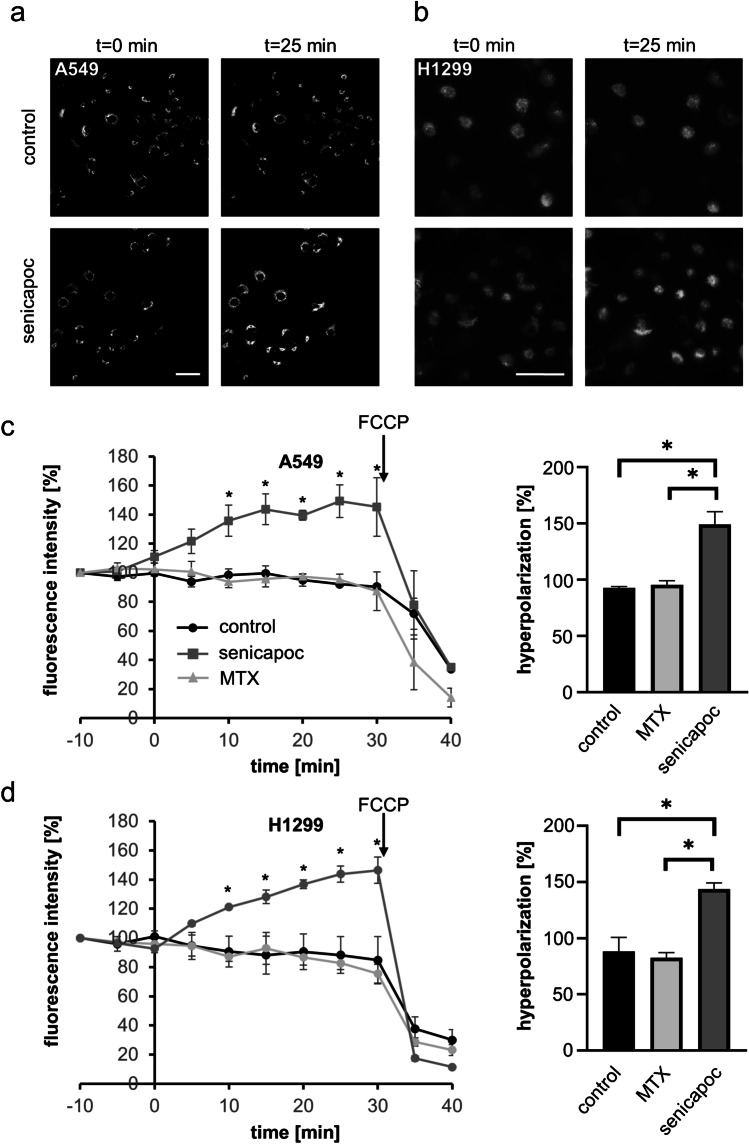
**The K**_**Ca**_**3.1 channel blocker senicapoc hyperpolarizes the mitochondrial membrane potential of NSCLC cells.** (**a**, **b**) Representative images of NSCLC cells stained with TMRM at different time points in the absence (control) and in the presence of senicapoc (scale bar: 50 µm). (**c, d**) Mitochondrial membrane potential measurements with A549 and H1299 cells. **Left figures,** senicapoc (30 µM), its solvent DMSO (1:1000 control) or maurotoxin (MTX; 20 nM) were added at *t* = 0 h. At the end of the experiments (*t* = 30 min), 1 µM FCCP was added. Data were normalized to fluorescence intensity at *t* =  − 10 min, and the curves represent the mean data from three different experiments (*N* = 3). **Right figures,** mitochondrial membrane potential determined at *t* = 25 min. For the statistical analysis, a two-way ANOVA of the mean data was performed, followed by Bonferroni’s multiple comparisons test. A *p* < 0.05 was defined as a statistically significant

Since the block of potassium fluxes across the inner mitochondrial membrane has been reported to result in hyperpolarization (e.g., Szabo et al. [42]), we measured the mitochondrial membrane potential in order to examine the function of K_Ca_3.1 channels in the inner mitochondrial membrane of NSCLC cells. The experiments were conducted using the cationic fluorescent dye tetramethylrhodamine methyl ester (TMRM). Figure 3a,b depicts representative micrographs obtained during these experiments. The measurements started with a 10 min control period. The mitochondrial membrane potential remained stable when the control solution was exchanged for one containing DMSO (1:1000), the solvent of senicapoc (ICA-17043), a high-affinity, membrane-permeant K_Ca_3.1 inhibitor [39]. Directly after adding senicapoc to A549 cells, the fluorescence intensity increases by up to 49.3 ± 11.2% which is indicative of hyperpolarization of the mitochondrial membrane potential (Fig. 3c**,** left panel). In contrast, the addition of the membrane-impermeant peptidic K_Ca_3.1 inhibitor maurotoxin that does not reach the inner mitochondrial membrane causes no change in the fluorescence intensity. This provides further evidence that the observed effects of senicapoc are due to inhibition of mitochondrial K_Ca_3.1 channels and not caused by an inhibition of channels in the plasma membrane. Each experiment was terminated by the application of the mitochondrial oxidative phosphorylation uncoupler FCCP which causes an immediate and complete depolarization of the mitochondrial membrane potential. FCCP also overrides the hyperpolarization of the mitochondrial membrane potential induced by the K_Ca_3.1 inhibitor senicapoc. The right panel of Fig. 3c

 summarizes the hyperpolarization at *t* = 25 min after the addition of the K_Ca_3.1 inhibitor senicapoc.

We also evaluated the role of K_Ca_3.1 channels in regulating the mitochondrial membrane potential of the NSCLC cell line H1299. In H1299 cells, senicapoc also increases the fluorescence of mitochondrial membrane potential indicator by up to 46% ± 9%. Thus, the mitochondrial membrane potential of H1299 cells hyperpolarizes in the presence of the K_Ca_3.1 channel blocker senicapoc as well. As for the A549 cells, the application of FCCP results immediately in a depolarization of the mitochondrial membrane potential in H1299 cells. As expected, neither DMSO nor maurotoxin changes the mitochondrial membrane potential of H1299 cells (Fig. 3d, left and right panels).

Taken together, pharmacological inhibition of K_Ca_3.1 channels affects the mitochondrial membrane potential in two NSCLC cell lines. Thus, our findings provide strong evidence for an important functional role of K_Ca_3.1 channels in the inner mitochondrial membrane of NSCLC cells.

### *The K*_*Ca*_*3.1 channel blockers senicapoc and TRAM-34 increase intracellular ROS levels in NSCLC cells*

**Fig. 4:**
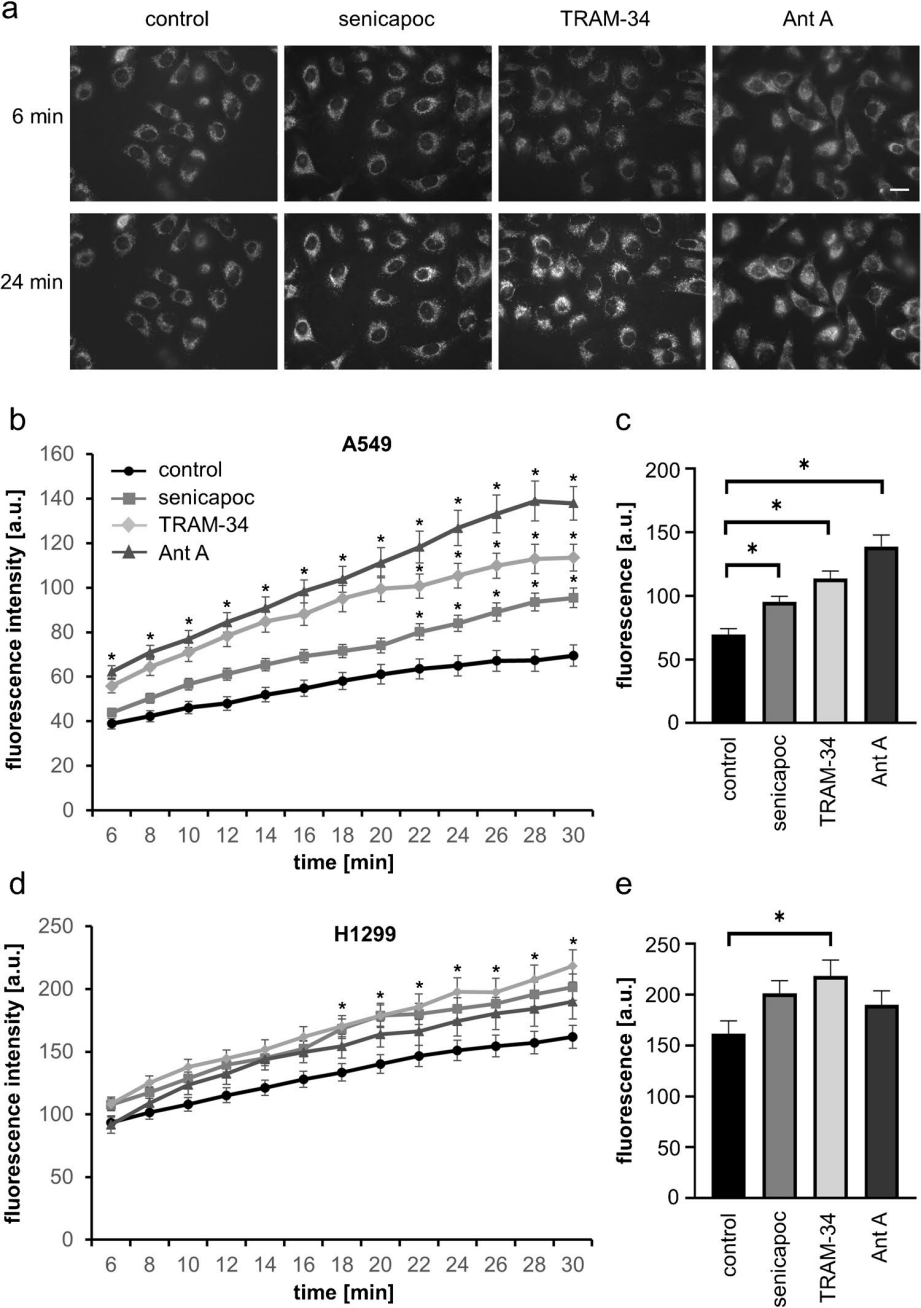
**The K**_**Ca**_**3.1 channel blockers TRAM-34 or senicapoc increase the production of superoxides (O**_**2**_^**−**^**) in NSCLC cells.** (**a**) Representative images of A549 cells stained with MitoSOX™ Red and acquired at *t* = 6 min and *t* = 24 min (scale bar: 20 µm). (**b**) Generation of O_2_^−^ in A549 cells treated with the K_Ca_3.1 channel blockers senicapoc (30 µM), TRAM-34 (10 µM), or the complex III inhibitor Antimycin A (2 µM) was recorded for a time period of 30 min. NSCLC cells were loaded with the mitochondrial superoxide indicator MitoSOX™ Red (10 µM) for 10 min. Antimycin A was used as a positive control for the generation of O_2_^−^. (**c**) Analysis of the endpoints (after 30 min) from each curve shown in Fig. 4b. Statistical analysis by using two-way ANOVA followed by Dunnett’s multiple comparisons test reveals a significant difference (*p < 0.05) between control conditions and the treatment with the K_Ca_3.1 channel blockers senicapoc or TRAM-34 (*N* = 4). (**d**) Generation of superoxide in H1299 cells exposed to K_Ca_3.1 channel blockers. (**e**) Analysis of the endpoints (after 30 min) from each curve shown in Fig. 4d. TRAM-34 induces the generation of O_2_.^−^ in H1299 cells (*N* = 4; two-way ANOVA, followed by Dunnett’s multiple comparisons tests; *p < 0.05)

To determine intracellular ROS levels in NSCLC cells, we applied the mitochondrial superoxide indicator MitoSOX™ Red to A549 and H1299 cells. The cells were exposed to senicapoc or TRAM-34 for a period of 30 min, and the increasing fluorescence intensity of MitoSOX™ Red was observed via fluorescence live-cell imaging. The measurements were validated by using the mitochondrial complex III inhibitor antimycin A which is known to induce the production of mitochondrial ROS in many cell types. The control solution contained DMSO (1:1000), the solvent for senicapoc, TRAM-34, and antimycin A. Figure 4a depicts original fluorescence micrographs revealing the increased fluorescence intensity of the treated cells at *t* = 24 min. Figure 4b displays the gradual increase of the fluorescence intensity in A549 cells with time. Initial fluorescent intensity at *t* = 6 min increases under control conditions from 38.9 ± 2.4 a.u. to 69.5 ± 4.8 a.u. at *t* = 30 min. In contrast, treatment with TRAM-34 (10 µM) leads to an increase from 55.8 ± 3.1 a.u. to 113.5 ± 6.0 a.u. and in the case of senicapoc (30 µM) from 43.9 ± 1.8 a.u. to 95.4 ± 4.3 a.u. Superoxide production is significantly higher after 22 min of treatment with the K_Ca_3.1 channel blockers. As expected, antimycin A (2 µM) elicits an even higher increase in superoxide production (from 62.2 ± 2.8 a.u. to 137.9 ± 7.5 a.u.).

The analysis of the last values (after 30 min) of each curve confirms the stimulatory effect of the K_Ca_3.1 channel blockers on mitochondrial superoxide production (Fig. 4c). These results indicate that the K_Ca_3.1 channel blockers TRAM-34 and senicapoc induce the production of superoxide anions (O_2_^−^) in A549 cells.

We next tested whether senicapoc and TRAM-34 also elicit the generation of ROS in another NSCLC cell line in H1299 cells. This is indeed the case, as shown in Fig. 4d,e. Here TRAM-34 and senicapoc are even more effective than antimycin A (218.4 ± 13.0 a. u. (TRAM-34) and 201.5 ± 10.6 a.u. (senicapoc) versus 161.9 ± 9.1 a.u. (control) or 190.1 ± 13.8 (antimycin A)). However, in H1299 cells, the effect of the K_Ca_3.1 channel inhibitors on ROS production is not as pronounced as in A549 cells.

Taken together, we have shown that the inhibition of mitochondrial K_Ca_3.1 channels with senicapoc or TRAM-34 increases the generation of mitochondrial superoxides in two NSCLC cell lines.

## Discussion

Earlier results showed the involvement of K_Ca_3.1 channels in numerous cellular processes of different cell types, including cancer cells. Our group previously found that K_Ca_3.1 channels contribute to several aspects of the metastatic cascade of NSCLC, including adhesion, migration, invasion, and proliferation [6, 7, 12]. Several other reports reinforced these findings also in other cancer cell types such as pancreatic, prostate, and endometrial cancer as well as glioblastoma (e.g., [15, 43]. In addition, it was also shown that pharmacological inhibition of K_Ca_3.1 channels with TRAM-34 causes a release of pro-apoptotic factors such as cytochrome c or Bax and thereby contributes to the apoptosis of melanoma cells [34]. That TRAM-34 increases intracellular ROS levels and induces apoptosis was also shown for hepatocellular carcinoma (HepG2) cells [25]. However, it did not become clear whether this was due to the inhibition of mitochondrial K_Ca_3.1 channels. Moreover, the direct demonstration of the expression of K_Ca_3.1 channels in mitochondria in HepG2 cells was missing.

Here, we revealed the functional expression of K_Ca_3.1 expression in the mitochondria of NSCLC cells. After demonstrating their mitochondrial expression on the protein level by means of Western blot and immunofluorescence, we also revealed their functional activity in the inner membrane of mitochondria. Both A549 and H1299 cells increase superoxide production in the presence of the K_Ca_3.1 inhibitors TRAM-34 or its analog senicapoc. Notably, the effect in H1299 cells was not as strong as in A549 cells. It is known that the tumor suppressor protein p53 is not expressed in H1299 cells which might have caused mitochondrial DNA depletion. This, in turn, could reduce the production of mitochondrial ROS [24]. This knowledge could also explain the only small effect of the complex III inhibitor Antimycin A. Depletion of p53 might alter mitochondrial complex III and thus modify the generation of superoxides [9].

In this context, it is also notable that H1299 cells had the lowest number of K_Ca_3.1 channels in the mitochondria of the cells tested in our study. This could be a further explanation for the reduced superoxide production in H1299 cells. However, a difference between H1299 and A549 cells could not be observed in the mitochondrial membrane potential measurements. In both cell lines, the K_Ca_3.1 channel inhibitor senicapoc elicits a strong hyperpolarization and thus shows a direct function of K_Ca_3.1 channels in the mitochondria. It has been shown previously that hyperpolarization of the mitochondrial membrane potential is accompanied by the generation of ROS, resulting in the release of cytochrome c that, in turn, triggers apoptosis [17, 33, 40, 48, 50].

Finally, we would like to discuss our method of determining the number of K_Ca_3.1 channel proteins. Of course, we are fully aware of the actual size of the K_Ca_3.1 channel protein which is about ~ 10 nm. Nonetheless, we are convinced that the fluorescent dots with an apparent diameter of ~ 300 nm represent single K_Ca_3.1 channel proteins. We refer to three of our previous papers in which we had developed a protocol using a dual-color quantum dot labeling approach for detecting and tracking single K_Ca_3.1 channel proteins [28, 29, 45]. The apparent size of ~ 300 nm for a fluorescently labeled single K_Ca_3.1 channel protein was derived from these studies. In addition, we would like to stress that the channel density derived from our labeling studies perfectly agrees with our earlier published patch clamp data [6]. Here, we measured the whole-cell current carried by K_Ca_3.1 channels in A549 cells. It amounts to 1600 pA at + 40 mV using a physiological K^+^ gradient. Considering the single channel conductance of ~ 25 pS for outward current and assuming an open probability of 50% (the pipette solution contained 1 µM free Ca^2+^), the whole-cell current could be generated by 3200 channel proteins. In the current manuscript, we determined the K_Ca_3.1 channel density in A549 cells to be 2.5 channels/µm^2^; 20% of this density was accounted for by mitochondrial channels. Thus, the density of K_Ca_3.1 channels in the plasma membrane is approximately 2/µm^2^. The surface area of A549 cells is ~ 1800 µm^2^ so that A549 cells have ~ 3600 channels in their plasma membrane.

In summary, our results show that pharmacological targeting of K_Ca_3.1 channels induces the generation of ROS in NSCLC cells. Interestingly, Western blotting revealed a slightly smaller molecular weight of K_Ca_3.1 channels in the mitochondrial fractions than in the whole cell lysates. This difference could be ascribed to proteolytic cleavage of a mitochondrial N-terminal targeting sequence after the import of the channel protein into the organelle. Alternatively, the whole cell lysates contain a glycosylated form of the channel protein, while it is unglycosylated in mitochondria. Just recently, a similar finding was demonstrated for mitochondrial K_V_1.3 channels [8]. Alternatively, it also could be that mitochondria expresses a splicing isoform of K_Ca_3.1 as it was found for mitochondrial K_Ca_1.1 channels in the heart [38]. Further studies would be needed to unravel the mechanism underlying the sorting of K_Ca_3.1 channels to mitochondria.

We included a figure as “Supplement Information”, containing representative blots detecting K_Ca_3.1, BAK and α-tubulin, as well as a corresponding description.
